# A dataset of multi-functional ecological traits of Brazilian bees

**DOI:** 10.1038/s41597-020-0461-3

**Published:** 2020-04-14

**Authors:** Rafael Cabral Borges, Kleber Padovani, Vera Lucia Imperatriz-Fonseca, Tereza Cristina Giannini

**Affiliations:** 1 Instituto Tecnológico Vale, Rua Boaventura da Silva 955, Belém, Pará Brazil; 2 Universidade Federal do Pará, Rua Augusto Correa 1, Belém, Pará Brazil

**Keywords:** Biodiversity, Zoology, Ecosystem ecology, Tropical ecology

## Abstract

Worldwide, bees are the most important group of animal pollinators. The ecosystem service they provide is vital in natural areas and croplands, and the taxonomic and functional diversity associated with bees is vital in understanding ecosystem functioning ensuring biodiversity conservation, food security and human livelihoods. A dataset of bees from mountainous areas of Carajás National Forest (eastern Amazon) and Nova Lima (Atlantic Forest) is presented here. It is a compilation of sampling efforts from 1983 to 2018 through the accession of data stored in museum collections. In total, 222 and 144 bee species were recorded in Carajás and Nova Lima, respectively. This represents the most robust dataset of Brazilian bees including species traits (body size, flight range, distribution, crop pollination, sociality and nesting) of 328 species. This dataset contributes to advances in the knowledge of the functional trait ecology of wild bees and can benefit further studies that analyze the response of wild bees to land use and climate changes, and its effects on the provision of crop pollination services.

## Abstract (summary)

**Table:** 

Measurement(s)	Trait • Ecology
Technology Type(s)	digital curation
Factor Type(s)	site
Sample Characteristic - Organism	Hymenoptera
Sample Characteristic - Environment	tropical broadleaf forest biome
Sample Characteristic - Location	Brazil

Machine-accessible metadata file describing the reported data: 10.6084/m9.figshare.12053742

## Background & Summary

Among insects, bees are the main pollinators for the majority of plants, being essential in both natural and crop environments for the provision of pollination services and for ensuring global food security to human population^1,2^. Additionally, these insects can be used as a means to improve human livelihoods, biodiversity conservation, and scientific, cultural and recreational development in natural, agricultural or urbanized landscapes^3,4^. In fact, the economic value of pollination services can be estimated for agricultural areas^5,6^. When considering natural areas, however, this value may be difficult to estimate, but this ecosystem function does influence local environmental quality, species conservation and the supply of native pollinators to crop fields^7–10^. Nevertheless, a global pollinator decline has been reported, mainly for managed species (e.g., *Apis mellifera* L.), but also for wild species (e.g., other bees, birds and bats)^11^, which directly affects the worldwide provision of pollination services^12^.

**Fig. 1:**
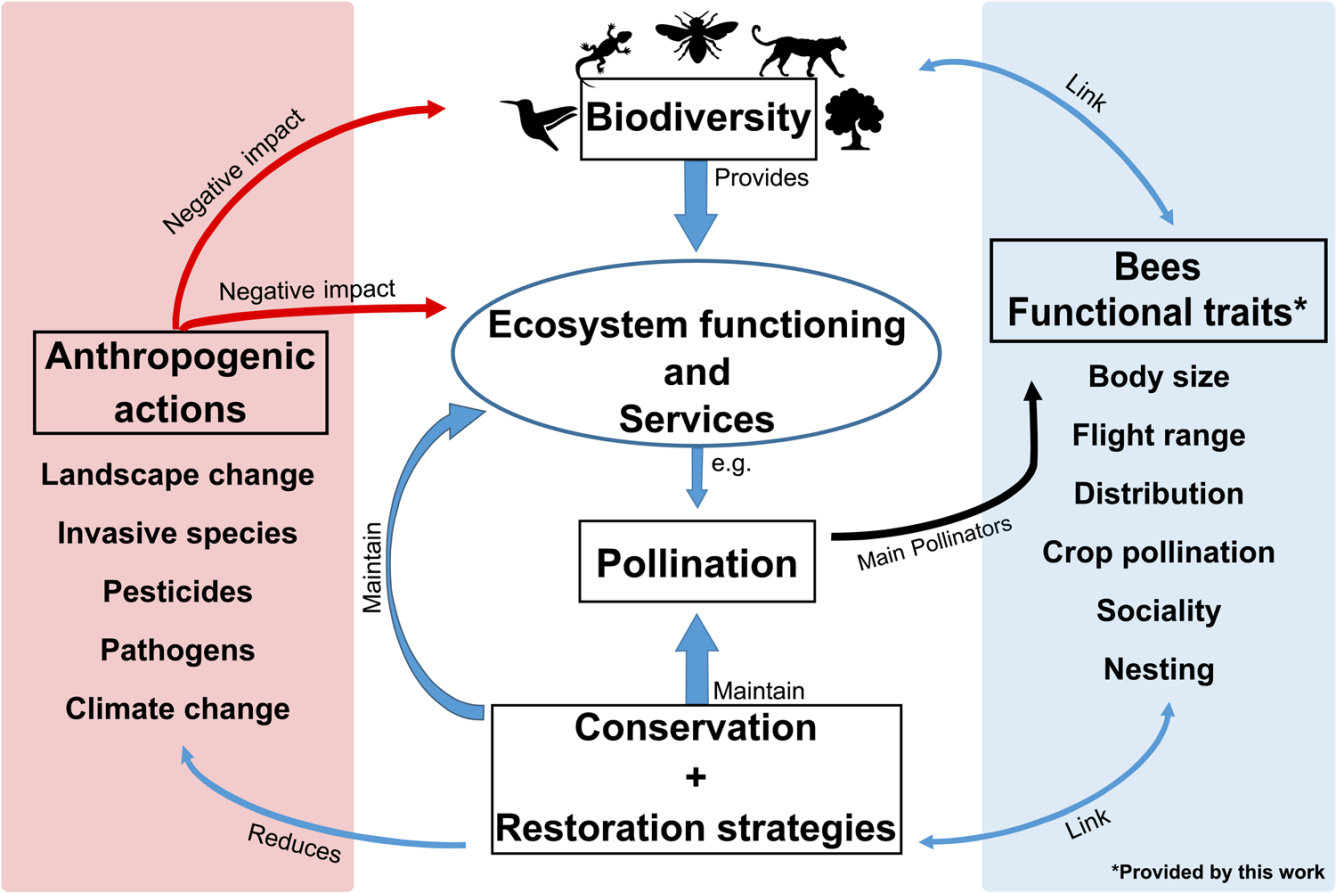
Graphical abstract showing the importance of the functional traits provided by this work and their relationship with studies on biodiversity conservation and ecosystem services provisioning.

Planetary decline and non-random loss of biodiversity have been reported in response to anthropogenic-driven actions^13^. Factors implicated in bee species decline include habitat loss due to landscape change, competition with invasive species for resources, emergent species (including pathogens), pesticides and climate change^12,14–16^. Linking ecosystem functioning to biodiversity conservation is fundamental in determining the aims of policies and strategies for species and ecosystem conservation in the long term^17^. In this context, the use of functional traits has arisen as a direct means of addressing the abovementioned link^18,19^ (Fig. 1).

**Fig. 2:**
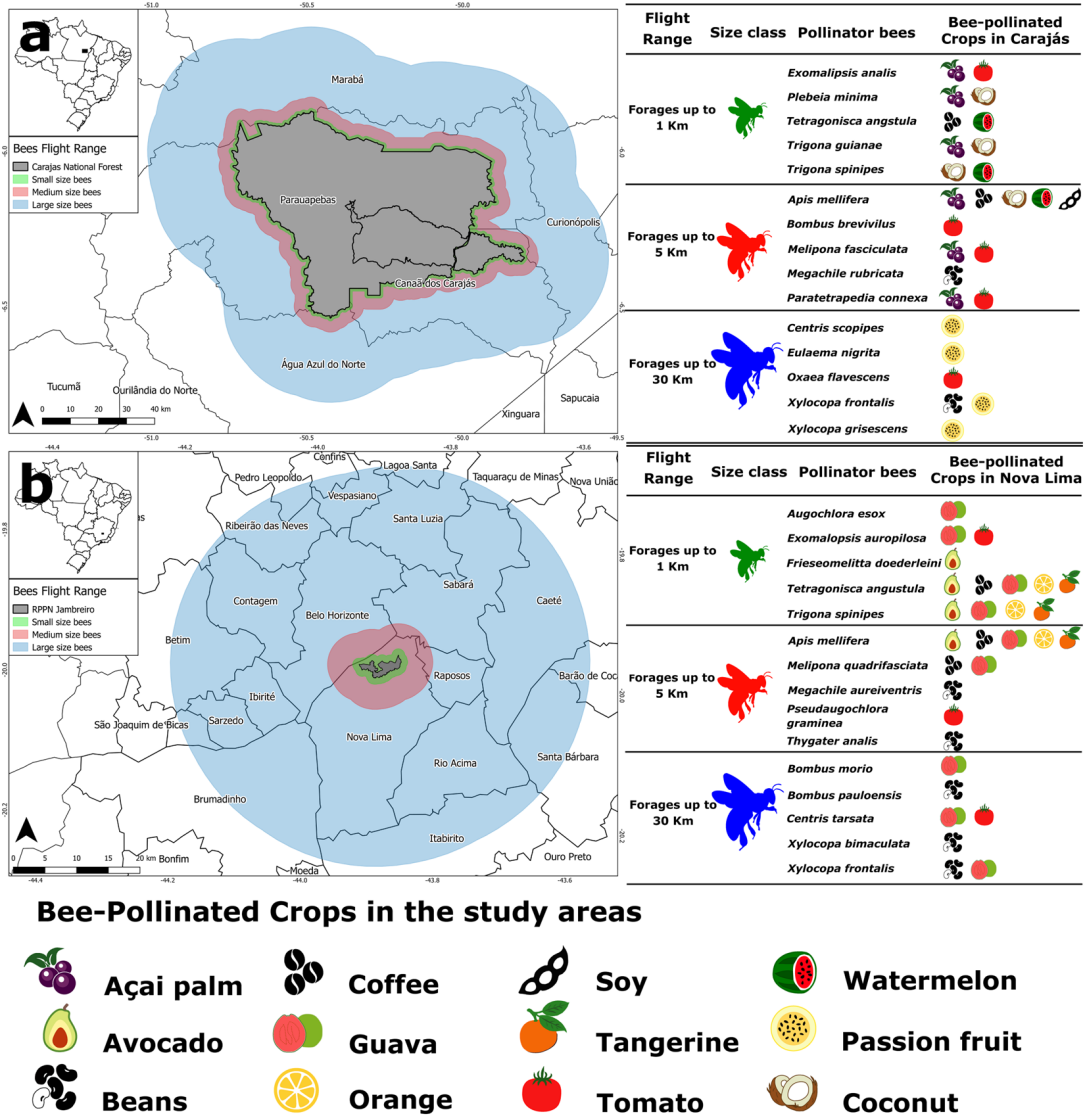
Flight range of each body size category with an overview of the main bee-pollinated crops and their main pollinators present in the study sites. (**a**) Carajás National Forest, eastern Amazon; (**b**) Nova Lima municipality, southeastern Atlantic forest.

Pollination success is related to pollinator species occurrence and availability^16^, but it also depends on the biological community assembly^17,20–22^ and on the relationship between flower traits (e.g., size and morphology) and the body size of its visitors^23^. Castilla *et al*.^24^ showed that pollinator body size contributes to plant seed viability but is apparently not related to long-distance genetic flow. Furthermore, pollination is a mobile agent-dependent ecosystem function that directly relies on the flight range capability of the pollinator agent^25^. For bee species, a positive relationship between body size and flight range has been well documented^26,27^, and foraging distance can determine the spatial scale within the landscapes at which bees are able to visit flowering plants^28,29^ (Fig. 2).

Ecosystem functioning relies mostly on a species’ ability to perform vital ecosystem functions^30^, and functional traits are the species characteristics that link them to their ecological function^31^. Bees are the major pollinator of plants because they directly depend on the supply of food provided by flowers (pollen and nectar)^32^. Floral rewards, however, are not the only requirement of bees to endure in the environment; they also require a nesting substrate and favorable landscape and climatic conditions^15,33^. Thus, our aim is to provide a database of species traits for Brazilian bees that are of significance to ecology and conservation^29,34,35^. Some important traits that explain bee community structure include body size, flight range, sociality, nesting location, nest behavior and diet^29,34,35^.

Here, we compiled a dataset of bee species from the Brazilian iron-rich mountains located in the Amazon (Pará State) and Atlantic Forest (Minas Gerais State). The dataset contains records of bees collected from 1983 to 2018 but in disconnected time frames. It represents one of the most comprehensive and robust datasets of bee species from Brazil and a unique dataset of bee species (including functional traits) from the Amazon forest, encompassing nearly 80% of the bee fauna from the eastern Amazon^36^. A total of 328 bee species records are provided here^37^. For those species, the following ecological traits are presented: body size, flight range, Neotropical distribution, crop pollination records, sociality and nesting substrate (Fig. 1).

## Methods

### Sites

#### Carajás national forest

**Fig. 3:**
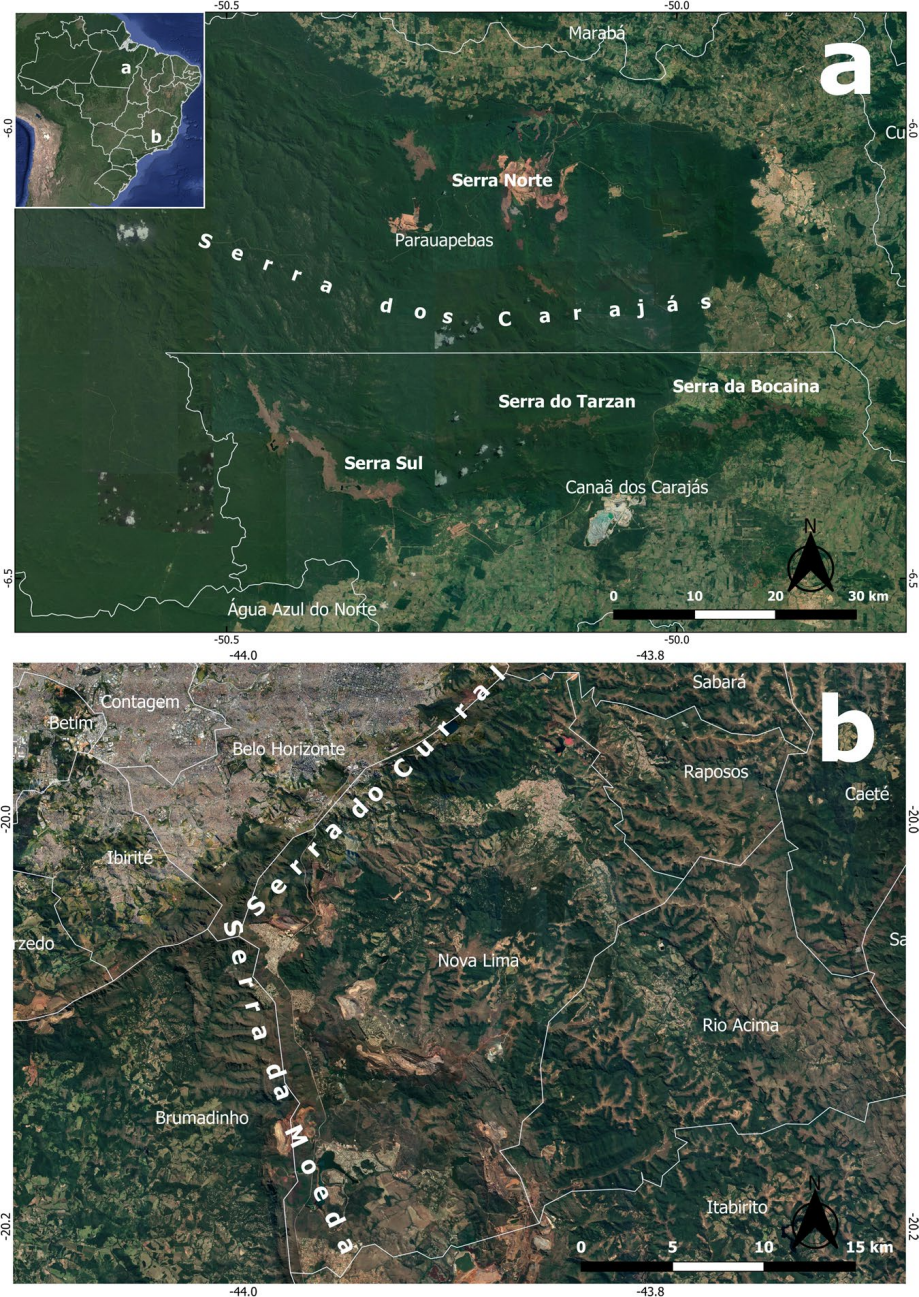
Study sites. (**a**) Serra dos Carajás in Pará State, eastern Amazon, northern Brazil. (**b**) Nova Lima in Minas Gerais State, Iron Quadrangle, Southeastern Brazil.

The Carajás National Forest (05°52′S–06°33′S, 49°53′W–50°45′W) is located in the southeastern portion of Pará State, Brazil (Fig. 3a). Carajás is an Amazonian domain area, mainly covered by forest formations^38,39^. It is located at an altitudinal range of 700 to 800 meters above sea level. The climate in this region is characterized as rainy tropical with winter drought (AWi), according to Koppen’s classification, with an annual precipitation range of more than 2000 mm and a well-defined drought period from June to September. The average temperature ranges from 25° to 26 °C, but the absolute recorded values range from 15 °C, from July to October, to 38 °C in the remaining months of the year. The predominant vegetation cover is evergreen ombrophilous forests; however, there are also areas of stationary vegetation with different degrees of deciduousness^39^. The largest Brazilian mineral extraction project is located in Carajás and deals primarily with the extraction of iron ore but also other minerals. It was initiated in the late 1980s and remains active to the present date.

#### Nova lima

Nova Lima is a Brazilian municipality in the state of Minas Gerais (Fig. 3b). Located in a mountainous landscape surrounded by the Serra do Curral and Serra da Moeda (altitude range: 900 to 1400 m), it is part of the region denominated as the “Plateaus and Mountain ranges of the East-Southern Atlantic” and is mainly composed of metamorphic rocks. The climate is hot and temperate and is classified as Cfa (subtropical humid climate). The average rainfall is 1390 mm per year, with August being the driest month and December the month with the highest precipitation. The average temperature is 23 °C; January is the warmest month of the year, and the lowest temperature occurs in June, i.e., an average temperature of 17.6 °C. The area of the municipality is 430 km^2^, and the population is estimated to be 92,000 inhabitants with a 0.8 HDI (human development index) (Brazilian Institute of Geography and Statistics - IBGE). The region has intense mineral extraction activities, including the mines of Morro Velho, Mostardas and Rio de Peixe, in addition to the recently closed mine of Águas Claras; the most important minerals are iron and gold. The city of Nova Lima integrates the metropolitan region of the state’s capital, Belo Horizonte (with approximately 2.5 billion inhabitants).

### Experimental and sampling design

The list of bee species names was obtained from two Brazilian entomological collections as the main repositories for specimens collected in both Carajás and Nova Lima, i.e., the Museu Paraense Emilio Goeldi (MPEG) entomological collection and the Universidade Federal de Minas Gerais (UFMG) entomological collection. Neither of these collections have online databases. Specimens of both collections were validated by specialists (for specimens’ IDs see^37^). Therefore, our dataset contains those records whose specimens were located and certified by specialists for both collections^37^.

At MPEG, we found bees from Carajás that were collected in the Serra Norte area (Fig. 3a) from 1983 to 2018. At UFMG, we found bees from Carajás that were collected in the Serra Norte and Serra Sul areas (Fig. 3a) during the 2008 to 2017 period. Bees from Nova Lima (Fig. 3b), collected from 1998 to 2017, were found only at UFMG. Bees were mainly collected with the use of entomological nets (active search), odoriferous traps (for male orchid bees, tribe Euglossini) and flight interception traps (malaise). Sampling efforts were not standardized, as a number of different researchers conducted their field work at these locations to answer several unrelated questions.

A total of 328 bee species have been recorded in these two areas, corresponding to more than 20% of the estimated 1500 bee species recorded in Brazil^40^. A total of 222 species were recorded in Carajás, representing nearly 80% of the bee fauna in Pará State, eastern Amazon (the 2^nd^ largest Brazilian state and the world’s 13^th^ largest state)^36^. More than 30% of these species are social (mostly the Meliponini tribe, but also the Bombini and Apini tribes), nesting mainly in pre-existing cavities (47%) or in the ground (22%), and 33% of these bees (at least at the generic level) have been identified as crop pollinators. A total of 144 species were recorded in Nova Lima, representing 65% of the bee fauna in Minas Gerais State (the 4^th^ largest Brazilian state)^36^. Nearly 80% of these species are solitary, more than 50% nest in the ground and 37% have been reported to be pollinators of Brazilian crops.

Although extensive sampling efforts were previously carried out in these areas, our dataset does not represent a final list but rather the consolidation of past sampling efforts together with species traits that are of great importance for ecological analysis and pollination services valuation^10^. New field sampling strategies are currently being carried out, following standardized methodology, to equalize sampling efforts and provide future comparison. Further steps will include a better understanding of the ecological functioning in these areas, with practical implications for the ecological restoration of mine-land rehabilitated areas in the Amazon Forest. Additionally, bee taxonomy for the Neotropical region is not complete, and recent taxonomic reviews continue to reveal new species, mainly from the less studied and most speciose areas, such as tropical rain forests^36,41,42^.

From this large dataset, it will be possible to answer more complex questions on the functioning of these ecosystems, such as the importance of the bees in each of these environments, the value of the ecosystem services they may provide and how these species will respond to ongoing global changes. Our database adds a robust set of trait-based information for Brazilian wild bees. We apply techniques that were previously reported in the literature^9,10,24,26,27^ and that are necessary for the advancement of functional ecology and the understanding of wild bees and their role in crop pollination and the provision of ecosystem services^28,29^.

### Traits acquisition and laboratory research methods

#### Body size, size classes and flight range

**Fig. 4:**
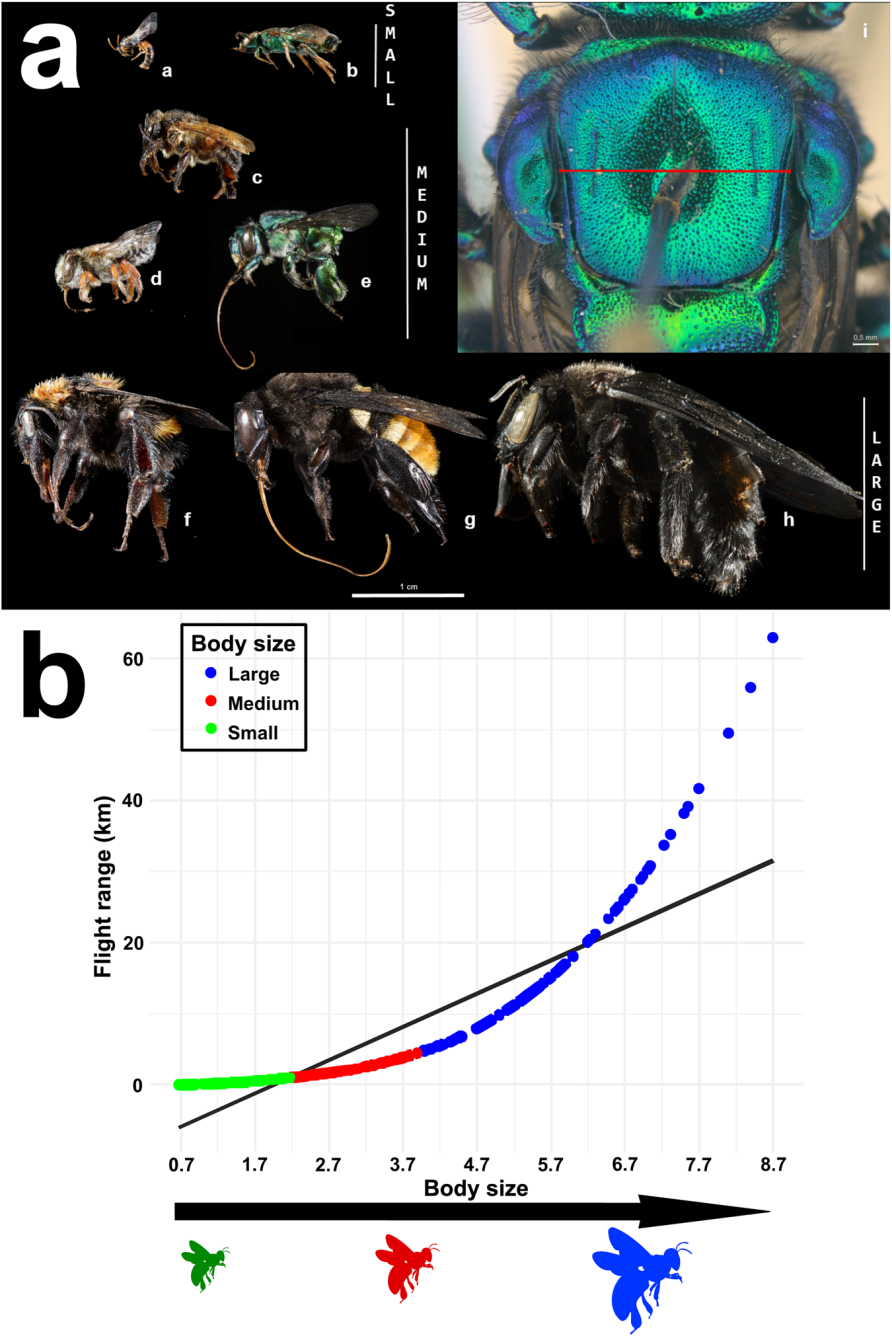
Bees body size and their relation to flight range. (**a**) Photos of bees showing their different body size classes. (**a**) Lateral view of *Hylaeus tricolor* (Schrottky, 1906) (**b**) *Augochloropsis callichroa* (Cockerell, 1900) (**c**) *Melipona seminigra* Moure & Kerr, 1950 (**d**) *Megachile orba* Schrottky, 1913 (**e**) *Euglossa amazonica* Dressler, 1982 (**f**) *Bombus transversalis* (Olivier, 1789) (**g**) *Eulaema cingulata* (Fabricius, 1804) (**h**) *Xylocopa frontalis* (Olivier, 1789). Photos by Fernanda Trancoso. (i) Dorsal view of a Euglossini bee with ITD (intertegular distance) measure. Photo by Rafael Borges. (**b**) Non-linear correlation between bees body size and flight range (estimated as the highest measurement obtained), dots represent bees in our data set and colors indicate body size categories (r² = 0.88).

Bees from the two abovementioned entomological collections were analyzed under a stereomicroscope coupled with a calibrated micrometric ocular. Body size measurements were based on the bees’ intertegular distance (hereafter ITD) (Fig. 4i). We measured up to five specimens of each species and the average value was calculated^37^. ITD measures represent the mesoscutum width of each bee species, which is the body section where the alary muscles are located. Bees with a larger ITD have been shown to be able to fly longer distances across landscapes^26^. The estimation of the flight distance of each bee species was calculated from the ITD measurement and taxonomic position using equations presented in^27^. Flight range estimations were based on models generated from previous on-site experiments for both social^43,44^ and solitary bees^45,46^. Recorded flight range experiments used two standard methodologies: (1) the release of marked bees at known distances from their nests and their recapture at the nest entrance (homing distance), considering the maximum homing distance (mhd – 90% return rate) and typical homing distance (thd – 50% return rate) and (2) feeder training techniques, which record the maximum energetically profitable foraging distance for a bee to forage at an artificial feeder (maximum foraging distance – mfd) and the distance to which bees stop recruiting nest mates to an artificial feeder (maximum communication distance – mcd)^26^.

We classified bees into three body size categories according to their ITD measures: small, medium and large (Fig. 4a). As body size and flight range are positively correlated, our body size categories also indicate maximum flight range, being up to 1 km for small bees, up to 5 km for medium bees and up to 30 km for large bees (Fig. 4b). Body size categories were established using the body size of bees in the genus *Melipona* Illiger as a standard for the medium-size class. Thus, ITD measures for the medium-size class ranged from 2.2 to 3.9 mm, with 2.2 mm being the ITD of *Melipona amazonica* Schulz, 1905, the smaller *Melipona* species in our dataset, and 3.9 mm being the ITD of *Melipona fuliginosa* Lepeletier, 1836, the larger *Melipona* species in our dataset. Bees with an ITD smaller than 2.2 mm were classified as small (e.g., *Trigonisca, Plebeia* and *Augochlora*), and bees with an ITD larger than 3.9 mm were classified as large (e.g., *Xylocopa, Centris* and *Eulaema*). Although arbitrary, our body-size classes are related to the fact that *Melipona* bees are commonly known as medium-sized bees.

Additionally, our classes agree with those previously established in other papers^24,47–50^ although some of those papers did not include *Melipona* bees. Therefore, we believe our classification is an appropriate standard for size classification of bees worldwide. To date, ours is the most species-rich dataset, which represents five bee families (Apidae, Andrenidae, Colletidae, Halictidae, Megachille) and includes the widest range of body size measurements reported. For example, the dataset includes the very large species *Xylocopa frontalis* (Olivier, 1789) (ITD = 8.4 mm) and also the minute *Trigonisca variegatifrons* Albuquerque and Camargo, 2007 (ITD = 0.6 mm).

#### Taxonomic ranks, known distribution, distribution area, new record, locality and location of measured specimens

Information on the taxonomic ranks (family, tribe, genus, subgenus, specific epithet, scientific name authorship) and known Neotropical distribution of each species were acquired using the online version of Moure’s bee catalog (available at http://moure.cria.org.br). Additionally, we searched and included updated information from the literature (after 2014, which was the last catalog update) when available. We chose to use Michener’s^32^ family classification instead of that provided in the catalog, as this classification is mostly used in other regions of the world besides Brazil. We included new occurrence records for all the specimens collected out of the known distribution provided in the Moure’s bee catalog.

Locality represents where each species was collected in our study sites. Localities from Carajás National Forest are Bocaina and Canaã dos Carajás from Serra Sul area and Carajás and Parauapebas from Serra Norte area. Remaining bee species were collected in Nova Lima.

Location of measured specimens indicate in which entomological collection (MPEG or UFMG) we found and measured the specimens collected at our study sites. For each of the specimens measured at the entomological collections (MPEG and UFMG) we provide the complete label information as well as specimen ITD measure^37^.

#### Crop pollination

Information on crop pollinator species was obtained from the database published by Giannini *et al*.^51^, which includes an extensive assessment of crop pollinators in Brazil. This database contains all the published interactions recorded between Brazilian crops and their pollinators, including the information source and the type of interaction recorded (effective, occasional or potential pollinator or simply a visitor).

In our dataset, we specify which species are known crop pollinators and also, for each bee species we specify which crop they were reported to pollinate^37^. Knowledge of crop pollinators is of great importance, not only in ecology and ecosystem functioning, but mainly for directing conservation strategies and policy decision making for ensuring food security. This knowledge can also be used as a means to evaluate ecosystem services provided by bees, to understand landscape population structure and to establish management and conservation strategies for individual species.

#### Sociality and nest location

Sociality and nest location data for each species were acquired by consulting previous natural history or review articles on these subjects. Primarily, a search for natural history data was performed for each species. For those species that completely lack natural history information, we searched for subgeneric information (when available), and when this information was not available, we included generic and tribe natural history information for the particular species. In all cases, before using generic, subgeneric and tribe information, a search in the specific literature was performed to verify the variation level in natural history traits for the specific clade.

We found sociality and nesting information for all species except some *Euglossa* Latreille, 1802 species. For 23 of the 43 *Euglossa* species, we did not find natural history data. Natural history traits vary widely within this genus, and the use of subgeneric or generic classification is not applicable for *Euglossa* species.

We provide the accuracy level of the information provided (i.e. tribe, genus, subgenus, species) and the reference papers from which natural history information was gathered for each species. The references are in the following format: authors, year, article title, journal, journal volume and pages^37^.

## Data Records

The complete database of species records, traits, measured specimen’s information and crops pollinated consists of 3 different files with descriptive names (Table 1). Files are all in ‘.csv’ (Unicode UTF-8) format and are stored in figshare^37^. Rows represent unique species records, and columns represent the variables provided (Online-only Table 1). For the 328 species, we present 438 trait records, 1530 specimens’ records and 932 crop pollination records. The dataset includes data for all five extant Neotropical bee families belonging to 77 genera.

**Table 1:** Summary information for the three data files compiling the information on the multifunctional ecological traits for Brazilian bees.

Data file name	N of bee species	Description	N rows	Ncolumns	File size
Brazilian_bees_traits.csv	328	Complete species traits file (body size, flight distance, known distribution, new occurrence records, crop pollination, sociality and nest location)	439	22	224.62 KB
Brazilian_bees_specimens_data.csv	328	Complete measured specimens data (location, label data, specimen collection code new occurrences data and sex information)	1531	19	197.27 KB
Brazilian_bees_crop_pollinators.csv	106	Complete list of crops pollinated	933	11	319.82 KB

We measured body size (from ITD, in millimeters) and categorized the mean species data into three body-size classes (small, medium and large).

Flight range, represented as the mhd, thd, mfd and mcd, was calculated from ITD using previously published flight distance estimation formulas^26,27^.

Specimens from two Brazilian entomological collections (MPEG and UFMG) were measured, and their location (Bocaina, Canaã dos Carajás, Carajás, Nova Lima and Parauapebas) was obtained from the specimen labels^37^.

The Neotropical distribution was obtained from Moure’s bee catalog (moure.cria.org.br). For species not included in the catalogue we used literature data and information from specimen’s labels. We provide new occurrence records from the specimens measured.

Crop pollination information was obtained from published plant-pollinator interactions compiled by Giannini *et al*.^52^ and is represented as yes (crop pollinator species) or no (not previously reported as a crop pollinator) in the traits file. We provide the reported interactions (each pollinated crop) for 106 bee species that pollinate 64 crops^37^.

Sociality and nesting information were obtained from 63 sources. Three categories of sociality were used: Cleptoparasitic; Eusocial; Solitary. Intermediary sociality classifications were not included as they would require further discussion and explanation, and this information is not the focus of this paper. All pillaging solitary bees (optional or obligate) were included as cleptoparasitic. Only eusocial bees (Apini, Bombini and Meliponini tribes that present obligatory cooperative brood care, division of labor, overlapping generations and reproductive castes) were considered Eusocial. The cleptoparasitic stingless bee genus *Lestrimelitta* Friese, 1903, was considered Eusocial. All the remaining species with different degrees of sociality were considered solitary. The level of accuracy for the acquired nesting and sociality information is provided based on the taxonomic rank for which the information was found available, being tribe, when information was found only for the species tribe, genus when information for the species genus was found, subgenus when information for the species subgenus was found and species when information for the species was found. Species with no available information, or when high taxonomic level information is not applied, are marked NA.

Nest location categories were based on the following ten classes:

**1. ant** – species that nest in association with a pre-existent ant nest;

**2. cavity** – species that nest in a pre-existent or excavated cavity in tree trunks or branches;

**3. cavity/human-made** – species that nest in a pre-existent or excavated cavity in human-made structures such as walls, bricks and fences;

**4. exposed** – species with exposed nests that are constructed around tree trunks or branches or around human-made structures;

**5. exposed/cavity** – species that nest in exposed areas and in cavities;

**6. soil** – species with subterranean nests in excavated or pre-existent cavities belowground;

**7. soil/cavity/human-made** – species that nest belowground and in pre-existent natural or human- made cavities;

**8. soil/cavity/termite** – species that nest belowground, in pre-existent cavities or in association with termite nests;

**9. soil/termite** – species that nest belowground, in association with subterranean termite nests;

**10. termite** – species that nest in association with pre-existent termite nests.

## Technical Validation

In our dataset we provide the list of bees from Carajás and Nova Lima that have been identified to species level by specialist taxonomists at both MPEG and UFMG entomological collections. This dataset is a compilation of the bee species collected in a long time frame in this areas (for sampling data see Borges *et al*.^37^). Locality and id (label data and entomological collection IDs) of measured specimens are provided to ensure they can be revisited. All measures were made by the same individual in order to reduce error. Information from literature was double checked and all sources are provided. For all available species we measured the ITD of up to five specimens in order to include intraspecific variation into our species body size measurements.

For estimating error rates, we manually re-collected data of 568 specimens from 122 species of our dataset (Supplementary Table 1). We found ITD measures differences on 26 out of the 568 specimens re-measured, which represents an error rate of about 4.5%. Errors varied from 0.1 to 0.5 mm; however, almost 70% of errors were distributed between 0.1 and 0.2 mm, which did not produced changes on average values. All the data for each specimen is provided (Brazilian_bees_specimens_data.csv^37^). We performed automatic and manual corrections and validation procedures for each dataset. All validation files, procedures and scripts are provided in Padovani and Borges^52^.

## Usage Notes

Note that ITD measures are presented as a medium value. We did this as a means to provide a representative value for the species, but a small intraspecific variation can also be found (see Brazilian_bees_specimens_data.csv^37^). Measured specimens’ data is provided ensuring the access to the individuals used in this database.

Traits regarding sociality and nesting location are all based on previously published papers. Note that for some species we rely on generic or subgeneric information in our database, so this can change once specific information are available, information on accuracy level is provided for each species.

### Supplementary information

Supplementary Table 1

## Data Availability

All validation files, procedures and scripts are provided in Padovani and Borges^52^. All files are available in Unicode (UTF-8) format.

## Online-only Table

**Online-only Table 1:** Definition of each variable included in the dataset.

Variable name	Variable definition	Units	Storage type
Id	Individual identification number for each sample.	N/A	Numeric
Family	The full scientific name of the family in which the taxon is classified	N/A	Character
Tribe	The full scientific name of the tribe in which the taxon is classified	N/A	Character
Genus	The full scientific name of the genus in which the taxon is classified	N/A	Character
Subgenus	The full scientific name of the subgenus in which the taxon is classified	N/A	Character
Specific epithet	The full scientific name of the specific epithet in which the taxon is classified	N/A	Character
Scientific name authorship	The person who was responsible for having written the textual scientific content of the original species description	N/A	Character
Body size class	Three body size classes estimated through ITDmeasured, as follows: large (ITD between 8.74 and 4 mm); medium (3.9 to 2.2 mm); small (2.1 to 0.7 mm)	N/A	Character
ITDmeasured	Mesoscutum width measurement. Species average calculated from measured specimens	millimeters	Numeric
Mhd	Flight range estimated as the maximum homing distance according to Cariveau *et al*.^25^	kilometers	Numeric
Thd	Flight range estimated as the typical homing distance according to Cariveau *et al*.^25^	kilometers	Numeric
Mfd	Flight range estimated as the maximum foraging distance according to Cariveau *et al*.^25^	kilometers	Numeric
Mcd	Flight range estimated as the maximum communication distance according to Cariveau *et al*.^25^	kilometers	Numeric
Location of measured specimen	Name of entomological collection where the measured specimens are deposited (MPEG stands for Museu Paraense Emilio Goeldi and UFMG for Universidade Federal de Minas Gerais)	N/A	Character
Known distribution	Species distribution known, quoted by Moure’s Bee Catalogue (moure.cria.org.br/)	N/A	Character
New record	Species for wich our measured specimens represent new occurrence records (yes or no)	N/A	Character
Locality	Locality in which the specimen was collected, being in Carajás National Forest (which includes Bocaina, Canaã dos Carajás, Carajás and Parauapebas) or in Nova Lima	N/A	Character
Crop pollinator	Species was quoted as crop pollinator by Giannini *et al*.^37^ (yes or no)	N/A	Character
Sociality	Species social organization as provided in the literature (Cleptoparasite, Eusocial, Solitary)	N/A	Character
Nest Location	Species nesting location in the landscape as provided in the literature (ant, cavity, cavity/human_made, exposed, exposed/cavity, soil, soil/cavity/human_made, soil/cavity/termite, soil/termite, termite)	N/A	Character
Level	Level of accuracy for Sociality and Nesting information (tribe, genus, subgenus, species)	N/A	Character
Ref.	References used to acquire sociality and nest location information	N/A	Character
Country	Country where the specimen was collected	N/A	Character
State	State where the specimen was collected	N/A	Character
Municipality	Municipality where the specimen was collected	N/A	Character
Day	Day when the specimen was collected	N/A	Number
Month	Month when the specimen was collected (in Roman numerals)	N/A	Number
Year	Year when the specimen was collected	N/A	Number
Location	Location where the specimen was collected	N/A	Character
Sampling point	Sampling site where the specimen was collected	N/A	Character
ITD	Specimen Intertegular distance	millimeters	Numeric
ITD average	Average ITD calculated from all measured ITDs for each species	millimeters	Numeric
Sex	Sex of each measured specimen (Male or Female)	N/A	Character
Collector	Name of the specimen collector	N/A	Character
Collection ID	Individual collection ID code	N/A	Numeric
Scientific name	Scientific name of each crop pollinator bee species	N/A	Character
Interaction Type	Type of interaction reported Giannini *et al*.^37^	N/A	Character
Plant	The full scientific name of the interacting plant taxon	N/A	Character
Vernacular name	Portuguese common name of the crop	N/A	Character
English vernacular name	English common name of the crop	N/A	Character
Pollinators ref	References used to acquire crop pollination information	N/A	Character
